# XPO1-inhibitor Selinexor induces MGMT expression by activating PKA-CREB signaling in *IDH* wildtype glioblastoma

**DOI:** 10.3389/fonc.2025.1633580

**Published:** 2025-09-23

**Authors:** Josephine A. Mapunda, Yuta Suzuki, Danielle Burgenske, Paul A. Decker, Lin Zhang, Jeanette E. Eckel-Passow, Jann N. Sarkaria, Gaspar J. Kitange

**Affiliations:** ^1^ Neuro-oncology Research, The Hormel Institute, University of Minnesota, Austin, MN, United States; ^2^ Department of Radiation Oncology, Mayo Clinic, Rochester, MN, United States; ^3^ Department of Quantitative Health Sciences, Mayo Clinic, Rochester, MN, United States; ^4^ Department of Public Health, University of Minnesota, Minneapolis, MN, United States

**Keywords:** temozolomide, resistance, XPO1, Selinexor, MGMT, glioblastoma

## Abstract

**Purpose:**

The temozolomide (TMZ) resistance mechanisms in MGMT-promoter methylated *IDH* wildtype glioblastoma (GBM) tumors are poorly known. This study aimed to identify potential modulators of TMZ resistance in methylated GBM cells.

**Methods:**

A genome-wide shRNA library screen was conducted to identify genes modulating resistance in a TMZ-resistant model of MGMT-methylated U251 GBM cells. The Incucyte Device was used for live cell growth monitoring, and DNA damage was assessed by foci staining.

**Results:**

*Exportin* (*XPO1*) was among the identified candidate TMZ-resistant genes, and the XPO1 inhibitor Selinexor was selected for further investigations. The MGMT-unmethylated GBM6 cells were sensitive to Selinexor alone, without additional sensitization when combined with TMZ. In contrast, MGMT-methylated GBM22 cells were relatively sensitive to Selinexor alone and were significantly sensitized to the Selinexor/TMZ combination. Interestingly, silencing MGMT sensitized GBM6 cells to the combined Selinexor/TMZ treatment, while forced exogenous MGMT expression blocked the sensitivity of U251 cells to the combined Selinexor/TMZ treatment. Selinexor treatment induced MGMT expression concurrently with increased phosphorylation of serine 133 of CREB protein (pCREB^S133^) in GBM6 and other MGMT-promoter unmethylated GBM cells. Finally, Selinexor-induced MGMT expression and pCREB^S133^ were blocked by the protein kinase A inhibitor H89, suggesting a role for PKA-CREB signaling in this process.

**Conclusions:**

This study demonstrates XPO1 as a mediator TMZ resistance in MGMT-methylated GBM cells, and that MGMT expression status is a potential determinant of sensitivity to Selinexor/TMZ treatment in GBM cells. These findings also uncover a novel mechanism linking Selinexor with PKA-CREB-mediated MGMT expression, suggesting that Selinexor may enhance MGMT-dependent TMZ resistance in GBM.

## Introduction

Glioblastoma, isocitrate dehydrogenase 1/2 (IDH1/2) wild-type (GBM) is an incurable disease with a poor patient median survival time of approximately 15 months and less than a 5% 5-year survival rate (1). GBM fatality is largely due to a high recurrent rate, often occurring within 6 months after the initial therapy, including surgery, radiation (RT), and temozolomide (TMZ) (1–3). The high rate of GBM recurrence is primarily due to the pre-existing (intrinsic) and secondary resistance to therapy, including radiation and TMZ therapy (4, 5).

Understanding the mechanisms of TMZ resistance is particularly important because it is the only FDA-approved first-line therapeutic agent for newly diagnosed GBM patients (6). The intrinsic TMZ resistance has been linked with the expression of DNA repair protein O6-methylguanine-DNA-methyltransferase (MGMT), and MGMT promoter methylation is a predictor of TMZ response (6–9). Unfortunately, all GBM patients with tumors lacking MGMT expression due to promoter hypermethylation eventually develop secondary TMZ resistance either due to re-expression of MGMT, loss of mismatch repair (MMR) proficiency, increased cellular potential to repair TMZ-induced DNA double-strand breaks (DSBs), or other unknown mechanisms (10). Thus, a better understanding of the mechanisms modulating secondary TMZ resistance could provide new molecular targets for developing novel effective therapies for MGMT-methylated GBM patients.

To comprehensively decipher the mechanisms driving resistance in MGMT-methylated GBM cells, we used a genome-wide shRNA library to identify candidate genes that may be targeted to overcome TMZ resistance in a TMZ-resistant U251(U251TMZ) model. We recently used a similar approach to identify and demonstrate RBBP4 as a modulator of primary TMZ resistance in GBM cells (11, 12). The current study used a resistant U251TMZ model to demonstrate that cells expressing the *Exportin 1* (*XPO1*) shRNA were resensitized to TMZ treatment. The *XPO1* gene encodes for a key protein that transports cargo from the nucleus to the cytoplasm through the nuclear pore (13). The XPO1 protein is an interesting target for cancer therapy because it plays a role in transporting tumor suppressor proteins from the nucleus to the cytoplasm (14–16). Indeed, the XPO1 inhibitor Selinexor is approved by the FDA for treating patients with refractory multiple myeloma (17, 18). Moreover, Selinexor is currently being evaluated as a treatment for patients with several human solid and liquid malignant tumors (15, 19–21). Relevant to this paper is a recent report suggesting a potential benefit of Selinexor in the treatment of patients with recurrent GBM (22). Nonetheless, like many newly identified anticancer agents, the molecular modulators of sensitivity to Selinexor and combined Selinexor/TMZ in GBM remain unelucidated. Here, we demonstrate that the MGMT-promoter methylated GBM cells are more vulnerable to a combined Selinexor/TMZ treatment and that Selinexor induces MGMT in promoter unmethylated GBM cells through activation of the protein kinase A- (PKA)-CREB pathway.

## Materials and methods

### Generation of TMZ-resistant model

The U251 TMZ-resistant model was established as previously reported (23). Briefly, mice with flank U251 tumors were treated with escalating oral doses of TMZ starting with 20mg/kg/day for 3 days, and after the initial tumor regrowth, mice were treated with 66mg/kg/day for another 3 days. The growth of the resulting resistant tumors was not inhibited by the highest TMZ concentration tolerable by mice, which was 120mg/kg/day for 5 days. TMZ-resistant flank tumors (hereafter named U251TMZ) were minced to a single-cell suspension and cultured as monolayers *in vitro*.

### Cell culture

Primary patient-derived xenograft (PDX) (GBM6, GBM43, GBM14 and GBM22) and the established GBM cells (U251, U251TMZ, and T98G) were cultured as previously described (24).

### shRNA library screening

The TMZ-resistant U251 (U251TMZ) cells were used for the shRNA screening of genome-wide modulators of TMZ resistance in MGMT promoter hypermethylated GBM cells. All the next steps were performed as we previously reported (11, 12). Briefly, cells were transduced with a pooled lentiviral shRNA (kindly provided by Dr. Yuichi Machida, Department of Oncology Research, Mayo Clinic, Rochester, MN) at a multiplicity of infection (MOI) = 1. This library targets about 38,000 genes or 47,000 mRNAs, and each shRNA is barcoded with the corresponding gene probe sequence used on the U133 plus 2.0 array (Affymetrix, Santa Clara, CA). After a brief selection in puromycin, cells were divided into 2 groups (each in triplicate). One group was treated with 100 µM TMZ while the other (control) group received the vehicle DMSO and was then allowed to grow for 14 days. Total RNA was extracted from control and cells survived TMZ treatment, followed by shRNA amplification according to a protocol supplied by System Biosciences (SBI, Mountain View, CA). The amplified shRNAs were hybridized onto the U133 plus 2.0 array (Affymetrix, Santa Clara, CA). The shRNAs with a significantly higher enrichment in the DMSO group in comparison to the TMZ group were considered to represent genes that negatively control TMZ sensitivity, and shRNAs preferentially enriched within the TMZ-treated group were considered positive modulators of TMZ sensitivity.

### Western blotting

Western blotting was performed according to the previously reported protocol (23). The primary antibodies included XPO1 (Exporitin-1/CRM1 (D6V7N) rabbit mAb, Cell signaling cat# 46249S), phospho-CREB^S133^ (87G3) rabbit mAb (Cell Signaling Cat# 9198S), CREB (48H2) rabbit mAb (Cell Signaling Cat# 9197S), anti-vinculin (E1E9V) XP(R) rabbit mAb (Cell Signaling cat# 13901S), beta-actin rabbit Ab (Cell Signaling Cat# 4967S), and secondary antibody was anti-Rabbit IgG, HRP-linked antibody (Cell signaling Cat. # 7074S).

### *In vitro* cytotoxicity assay

Primary and established cells were plated in 96-well plates (primary, 2000/well; established, 500/well) and maintained at standard humidified culture conditions (37°C and 5% CO_2_) overnight. Then, cells were exposed to graded concentrations of Selinexor, TMZ, and combined Selinexor/TMZ. The control cells were treated with DMSO. The growth of the control and treated cells was live monitored using an Incucyte SX5 machine (Sartorius Inc., Ann Arbor, MI) or by the Cyquant Cell proliferation assay (Thermo Fisher Scientific, Cat# C7026). All experiments were conducted in triplicate and replicated three times.

### Gene knockdown and re-expression

Lentiviral shRNA constructs were used for gene knockdown. Briefly, the pLKO shRNA constructs were purchased from Millipore-Sigma (Danvers, MA). Lentiviral particles were packaged using the 293T cells. For this, the 293T cells were plated into 100mm culture dishes to be about 70-80% confluent the next day. After overnight attachment, cells were changed to antibiotic-free media, and the co-transfection of plasmids was done by diluting 54 μL of FuGENE6 (Roche) in 2 mL of serum-free DMEM and incubating for 5 minutes at room temperature. Then, Gag-Pol (3ug), VSG (3ug), and pLKO shRNA (4.5ug) plasmids were added, mixed by flicking, and incubated for 20 minutes at RT, and then added into cells. The day after the infection, 10 mL of antibiotic-free fresh media was added to the dishes, and 2 days later, the media were collected, briefly centrifuged to collect the cell debris, filtered through 22-um filters, aliquoted, and stored at -80°C until use. For MGMT re-expression experiments, MGMT cDNA was cloned into a GIPZ lentiviral plasmid (Horizon Discovery, Lafayette, Colorado) to replace the GFP-cDNA. Both empty vector (GIPZ-GFP) and GIPZ-MGMT plasmids were packaged using the above-described protocol for shRNA constructs. The packaging and handling of the lentiviral particles were done following the University of Minnesota Biosafety Committee guidelines.

### γ-H2AX DNA damage foci

U251 cells expressing empty vector (U251V) and MGMT cDNA (U251 MGMT) were grown overnight on coverslips. Cells were treated with TMZ or vehicle DMSO and fixed with 4% paraformaldehyde at 0 and 72-hour time points. Irradiated cells (2-Gy) were used as a positive control for γ-H2AX. The immunofluorescence staining was conducted as previously described (11). The primary antibody used was P-Histone H2A.X (S139)(20E3) rabbit mAb (Cell Signaling Cat# 9718S), and the secondary antibody was Alexa Fluor™ 594 goat anti-rabbit IgG (H+L) (Invitrogen Cat# A11012). The staining was analyzed with a confocal microscope (Zeiss LSM 900 with Airyscan detector, Zeiss, Germany). For γ-H2AX foci quantification, at least 200 cells with ≥ 25 foci/nuclei were analyzed for each condition.

### Chromatin immunoprecipitation

Chromatin immunoprecipitation (ChIP) was performed using the MAGNA-ChIP™ kit, Cat. # 17-10085(Millipore, Billerica, MA). Cells were crosslinked with 1% formaldehyde, and after quenching the formaldehyde reaction with 0.1 M glycine, the cells were processed according to the protocol supplied by the vendor (Millipore). Antibodies used were anti-acetyl-lysine 27 histone H3 (H3K27ac rabbit monoclonal antibody, diagenode Cat# C15210016) and anti-trimethyl-lysine 4 histone H3 (H3K4-me3,1, Epigentek Cat# P-2028-24). The distal promoter region that is critical for MGMT silencing by hypermethylation (25) was targeted by a quantitative PCR using human-specific primer sequences: 5′-GCCCCGGATATGCTGGGAC-3′ (forward) and 5′-GGGCAACACCTGGGAGGCAC-3′ (reverse). ChIP enrichment was evaluated relative to the input chromatin.

### Quantitative RT-PCR

RNeasy Kit was used for total RNA extraction from GBM cells according to the protocol supplied by the vendor (Qiagen Inc., Germantown, MD). The reverse transcriptase was performed as previously reported (23, 25). The following primers were used to amplify MGMT 5’ TCT TCA CCA TCC CGT TTT CC-3’(forward) and 5’-CCG AAT TTC ACA ACC TTC AGC-3’ (reverse); XPO1 5’-CTA CAT CTG CCT CGT TGCT-3’ (forward) and 5’-CCA ATA CTT CCT CTG GTT TAG CC-3’ (reverse); GAPDH 5’-CTC TGC TCC TGT TCG AC-3’ (forward) and 5’-GCC CAA TAC GAC CAA ATC C-3’ (reverse). The PCR mix was prepared using PowerUP™ SYBR™ Green Master Mix (Applied Biosystems Cat# A25742), and the PCR reaction was performed at 95°C for 10 minutes, followed by 40 cycles of 95°C denaturing, 60°C annealing, 72°C extension, and final extension at 72°C for 10 minutes.

### Statistical analysis

Linear mixed models were used to compare the rate of % confluence changes over time between treatment groups, followed by pairwise comparisons using the Tukey method. The differences in percent confluence in Selinexor with and without TMZ and the ChIP enrichment were analyzed using a two-sample t-test. The p-values <0.05 were considered statistically significant.

## Results

### XPO1 modulates TMZ resistance in GBM cells

**Figure 1:**
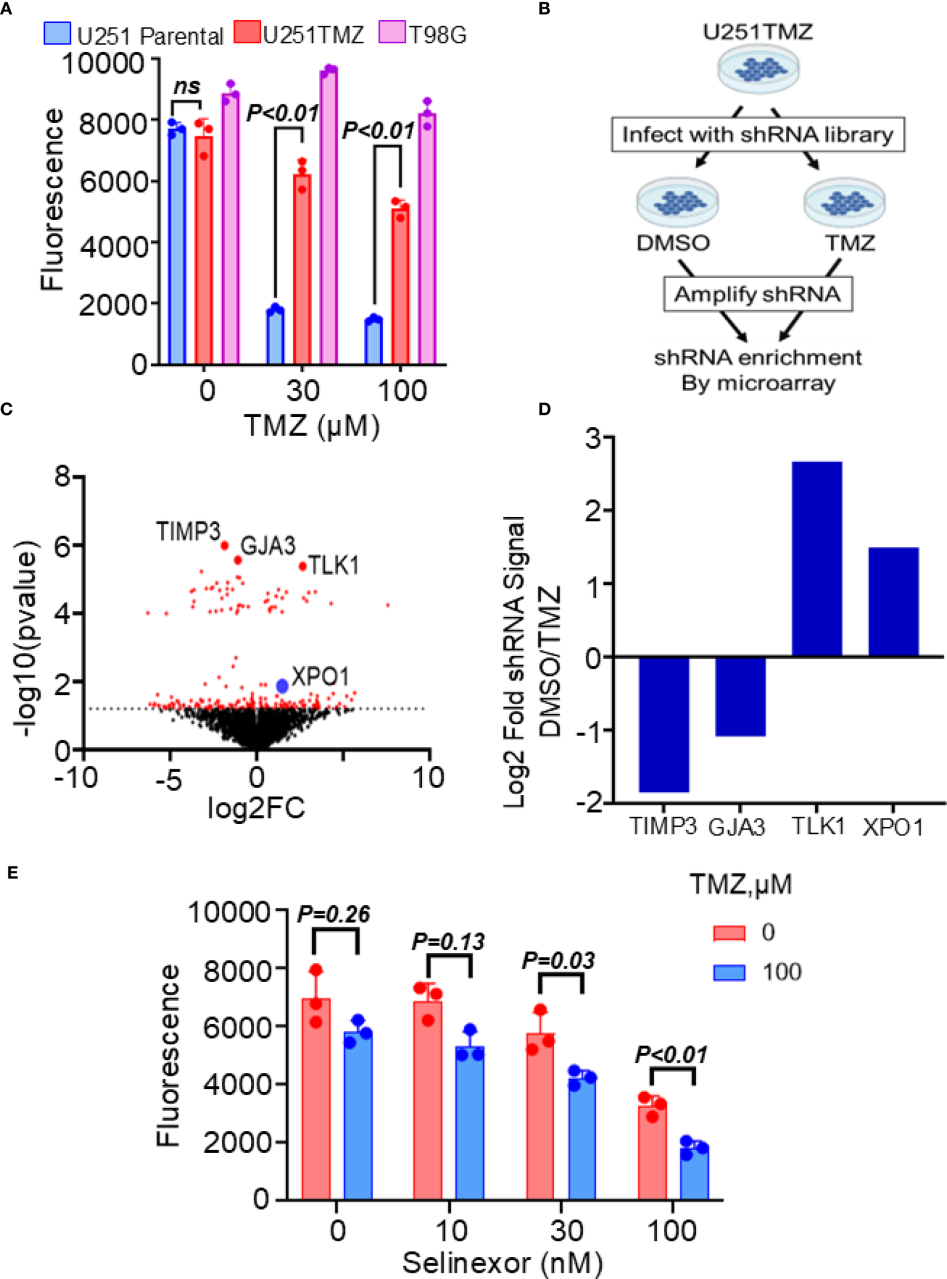
Effects of XPO1 and the inhibitor Selinexor on TMZ sensitivity in U251TMZ resistant model. **(A)** The U251TMZ resistant model was generated *in vivo* by treating mice carrying U251 flank tumors with an escalating dose of TMZ. Cells cultured from tumors that continued to grow in the presence of the highest concentrations of TMZ but were tolerable to mice (120 mg/kg) were grown in culture and evaluated for TMZ sensitivity compared to the parental placebo-treated mice. The MGMT-expressing TMZ-resistant T98G cells were used as a positive control. **(B)** Graphical display of the whole genome shRNA library used to identify genes associated with the evolution of resistance in the U251TMZ model. **(C)** Volcano plot showing shRNA enrichment comparing the DMSO control *vs*. the TMZ-treated U251TMZ cells. **(D)** Bar graph displaying representative shRNA enrichment in TMZ-treated relative to DMSO-treated cells. **(E)** U251TMZ cells were treated with different concentrations of XPO1 inhibitor Selinexor with and without 100 µM TMZ and growth was monitored using a Cyquant cell proliferation assay. Data represent mean ± SEM from n = 3 independent biological replicates, each performed in technical triplicates. Statistical significance was determined using a two-way ANOVA with Tukey’s multiple comparison test (ns, not significant).

We developed a resistant model of U251 cells to study the mechanisms modulating TMZ resistance in MGMT-promoter hypermethylated GBM cells. Unlike previous models created by treating cells with TMZ *in vitro*, our U251-resistant model was generated *in vivo* by treating mice harboring flank U251 xenografts with escalating doses of TMZ until reaching the maximum dose tolerated by mice, but without any further slowing down the tumor growth. As shown in **Figure 1A**, cells cultured from these flank tumors (referred to hereafter as U251TMZ) were significantly resistant to TMZ compared with the parental U251 cells (relative fluorescence (30 µM TMZ) = 1803 ± 91.00 (parental) vs. 6239 ± 465.5 (U251TMZ); p-value = 0.01). To uncover the molecular modulators of TMZ resistance in U251TMZ, we performed the whole genome shRNA library screening as graphically displayed in **Figure 1B**. Using this approach, we identified shRNA candidates that, when expressed, either enhance or suppress the sensitivity of U251TMZ cells to TMZ, as shown by a volcano plot. (**Figure 1C**). We focused on the shRNA candidates preferentially enriched in the control DMSO-treated over TMZ-treated cells, indicating that the genes targeted by these shRNA are negative modulators of TMZ sensitivity in U251TMZ cells. Interestingly, the nucleocytoplasmic protein XPO1 was among the top 25 genes with the shRNA that resensitized U251TMZ cells to TMZ, as indicated by increased enrichment of XPO1 shRNA in DMSO-treated cells (**Figure 1D**). Since the XPO1 inhibitor Selinexor is a brain-penetrant compound and a potential therapeutic agent for GBM therapy (22, 26), we evaluated whether this compound could overcome resistance in U251TMZ cells. As shown in **Figure 1E**, Selinexor significantly resensitized U251TMZ cells to TMZ (Relative fluorescence: TMZ (100 µM) alone = 5820 ± 392.8 and Selinexor (100 nM) alone = 3273 ± 341.1 vs. TMZ (100 µM)/Selinexor (100 nM) = 1828 ± 231.4; p-value < 0.001). Together, these findings suggest that XPO1 may contribute to the evolution of TMZ resistance in MGMT-promoter hypermethylated GBM cells, and Selinexor can potentially be used for overcoming TMZ resistance.

### MGMT expression influences sensitivity of GBM cells to Selinexor/TMZ treatment

**Figure 2:**
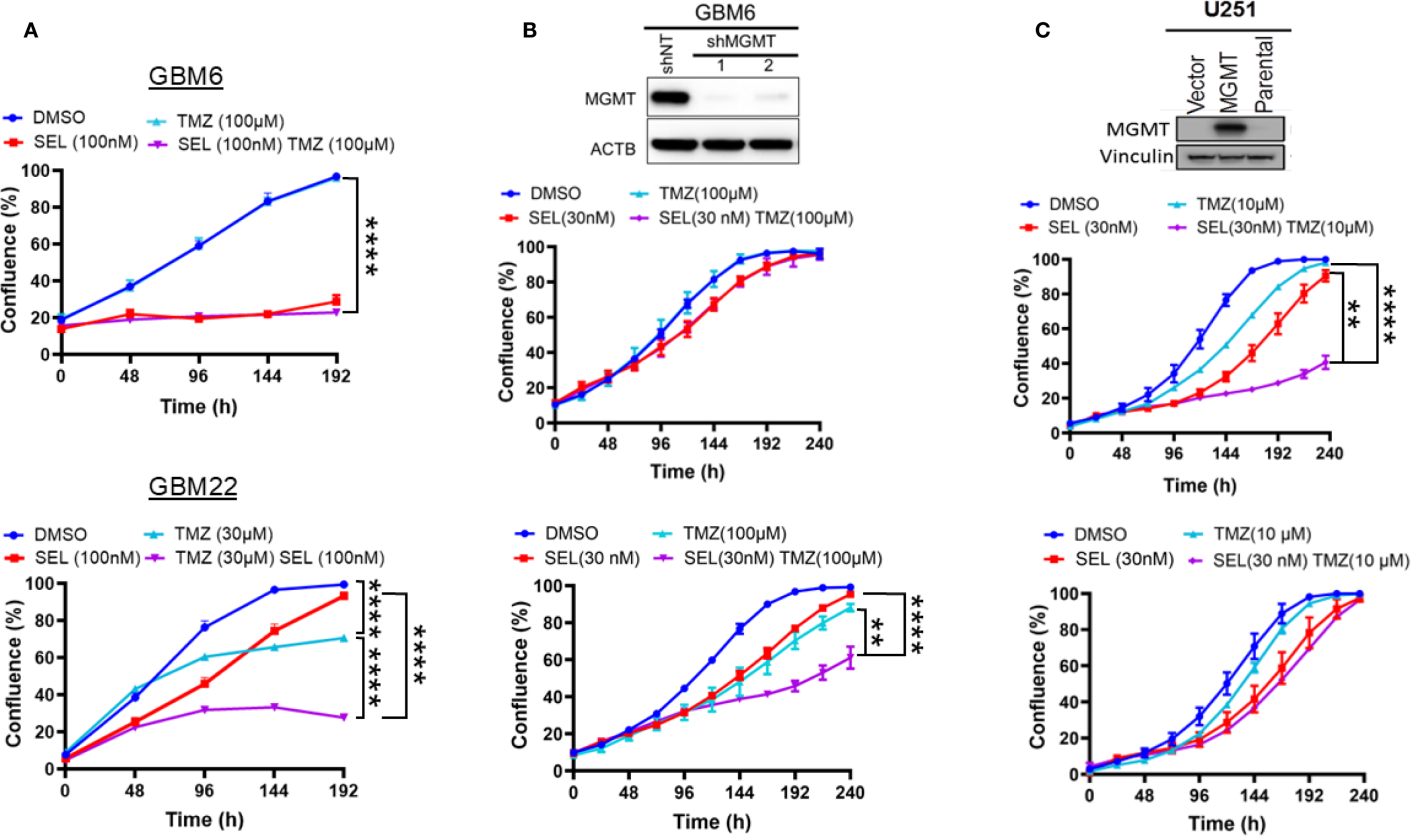
Influence of MGMT expression status on the sensitivity of GBM PDX cells to a combined SelinexorTMZ treatment. **(A)** Primary cells cultured from MGMT expressing GBM6 (upper panel) and MGMT null GBM22 (lower panel) were plated in 96-well plates and treated with the indicated concentrations of Selinexor, TMZ, and Selinexor+TMZ and growth was monitored using an Incucyte Live Cell monitoring device and reported in percent confluence. **(B)** GBM6 cells were infected with control shNT and two MGMT shRNA lentiviral constructs. After selection, the effective knockdown was determined using western blotting (upper panel) and both GBM6 shNT (middle panel) and GBM6shMGMT (lower panel) were treated with the indicated concentrations of Selinexor, TMZ, and TMZ + selinexor, followed by live growth monitoring using an incucyte device. **(C)** Forced exogenous MGMT expression in MGMT-methylated U251. The MGMT-negative U251 cells were transfected with a control empty vector (U251V) and MGMT cDNA vector (U251MGMT). After confirming the expression by western blotting (upper panel), the U251V (middle panel) and U251MGMT (lower panel) cells were treated with the indicated concentration of selinexor, TMZ, and TMZ + Selinexor. Growth was monitored using an incucyte device for the indicated time. Data are presented as mean ± SEM from n = 3 independent biological replicates, each performed in technical triplicates. Statistical analyses were performed using a two-way ANOVA with Tukey’s multiple comparison test to compare treatment groups. Significance is denoted as **p-value <0.01, ****p-value <0.001.

The above findings suggest that Selinexor may control the sensitivity of MGMT-methylated GBM cells to TMZ. Since MGMT expression plays a critical role in the sensitivity to TMZ (6, 8, 9, 27), we evaluated whether the expression of MGMT influences the sensitivity of GBM cells to the combined Selinexor/TMZ treatment. To this end, we used cells primarily cultured from two GBM PDXs (MGMT-unmethylated GBM6 and MGMT-methylated GBM22) and two established GBM cell lines (MGMT-unmethylated T98 and MGMT-methylated U251). Expectedly, the MGMT-unmethylated GBM6 cells were resistant to TMZ (DMSO vs. TMZ 100 µM p-value = 0.17) but highly sensitive to Selinexor alone (DMSO vs. Selinexor p<0.0001), and there was no additional growth suppression by combining TMZ with Selinexor (p-value = 0.16; **Figure 2A**; upper panel). Similar results were observed in the established MGMT-unmethylated T98G cells (**Supplementary Figure S1**). In contrast, the MGMT-methylated GBM22 cells were sensitive to TMZ (30 uM), and this sensitivity was significantly enhanced by combining Selinexor with TMZ (DMSO vs. TMZ p<0.0001; TMZ vs TMZ/Selinexor p<0.0001; **Figure 2A**; lower panel). These findings closely resemble the data showing that siRNA silencing of XPO1 enhances the sensitivity of U251 cells to TMZ (**Supplementary Figure S2**). Unexpectedly, MGMT-unmethylated GBM6 cells demonstrated a higher sensitivity to Selinexor (**Figure 2A**; upper panel) than the MGMT-methylated GBM22 cells, which initially showed a diminished proliferation in response to Selinexor alone but progressively recovered (**Figure 2A**; lower panel), suggesting that GBM22 cells may be less vulnerable to Selinexor inhibition than GBM6 cells. Indeed, we found that Selinexor (100–1000 nM) completely inhibited the XPO1 protein in GBM6 cells, while similar concentrations did not fully block the expression of XPO1 protein in GBM22 (**Supplementary Figure S3**).

To confirm that MGMT status determines the sensitivity to Selinexor/TMZ treatment, we reexpressed the exogenous MGMT in promoter methylated U251 (U251MGMT) or the control empty vector-expressing U251V (**Figure 2C**, top panel). Interestingly, the control U251V cells were sensitive to either single-agent TMZ treatment (10 µM) or Selinexor (30 nM). A significant increase in sensitivity was observed when TMZ was combined with Selinexor (p<0.001; **Figure 2C**, middle panel). In contrast, U251MGMT cells were resistant to TMZ and slightly sensitive to Selinexor alone, but no additional effect on proliferation was observed by combined TMZ/Selinexor treatment (**Figure 2C**, lower panel). To further demonstrate the role of MGMT in selinexor/TMZ sensitivity, we knocked down MGMT in promoter-unmethylated GBM6 cells (**Figure 2B**, upper panel). MGMT-depleted GBM6 cells showed a relatively increased sensitivity to Selinexor, which was further potentiated when combined with TMZ (DMSO *vs*. Selinexor p<0.01, DMSO *vs*. TMZ, p<0.001; TMZ *vs*. TMZ/Selinexor, p<0.001, Selinexor *vs*. TMZ/Selinexor, p<0.0001; **Figure 2B**, lower panel) compared to control shNT-expressing cells (**Figure 2B**; middle panels), further highlighting the potential role of MGMT in modulating responsiveness to Selinexor/TMZ treatment.

**Figure 3:**
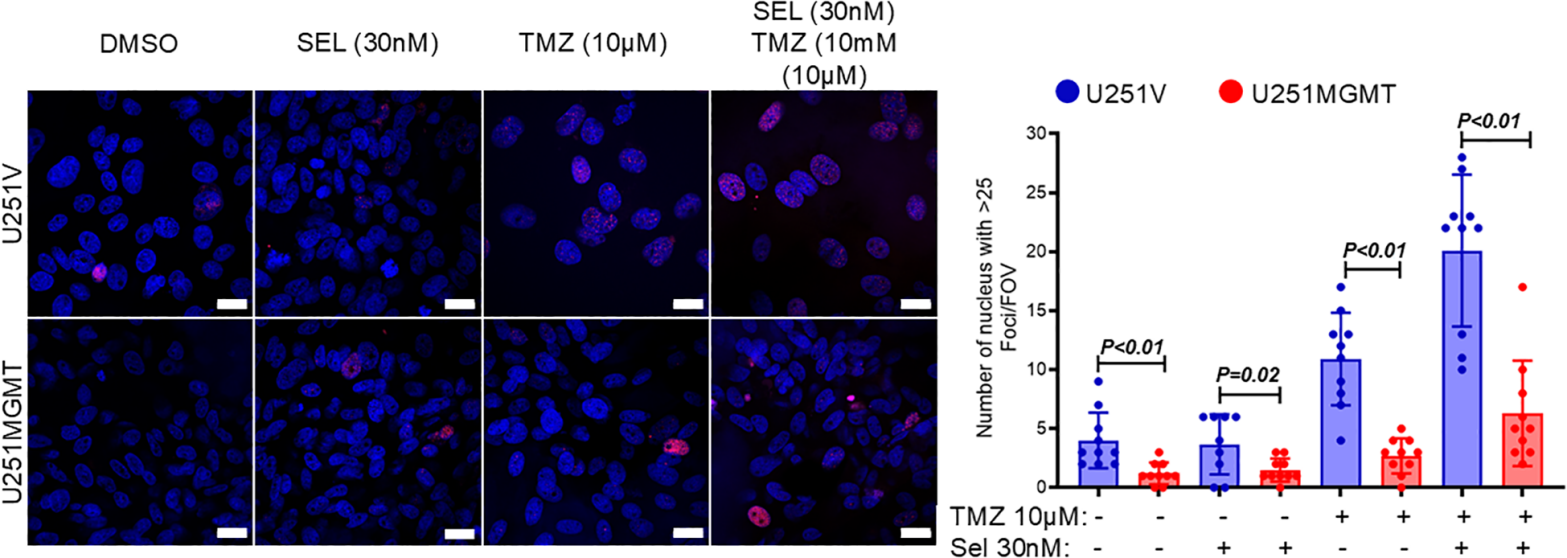
Evaluation of γH2AX foci in U251V and U251MGMT treated with Selinexor with and without TMZ. Cells were treated with the indicated concentrations of TMZ, Selinexor, and Selinexor + TMZ, and γH2A foci were detected using immunofluorescence staining performed 72 hours later (left panel). The number of nuclei with ≥ 25 γH2AX foci was counted and graphed (right panel). Magnification bar = 20µM. Data are presented as mean ± SEM from n = 3 independent biological replicates, with at least 100 nuclei analyzed per condition in each replicate. Statistical significance was determined using a two-way ANOVA with Tukey’s multiple comparison test. Error bars represent SEM.

In parallel with suppressing the sensitivity to selinexor/TMZ treatment, exogenous MGMT significantly diminished the Selinexor-mediated potentiation of TMZ-induced DNA double-strand breaks (DSBs) as evidenced by decreased ɣ-H2AX foci both in TMZ- and Selinexor/TMZ-treated U251MGMT compared with the control U251V (**Figure 3**, left panel). The number of nuclei with ≥ 25 ɣ-H2AX foci in Selinexor/TMZ-treated U251MGMT was 6.30 ± 1.415, whereas for Selinexor/TMZ-treated control U251V was 20.10 ± 2.036, a statistically significant difference (p<0.0001). Consistent with the increased TMZ-induced DNA damage, Selinexor-potentiated TMZ-induced PARP-cleavage indicated an increased cell death through apoptosis in U251V but not in U251MGMT (**Supplementary Figure S4**). Together, these findings support the notion that MGMT may influence the sensitivity to combined TMZ/Selinexor treatment.

### Selinexor induces MGMT expression in unmethylated GBM cells

**Figure 4:**
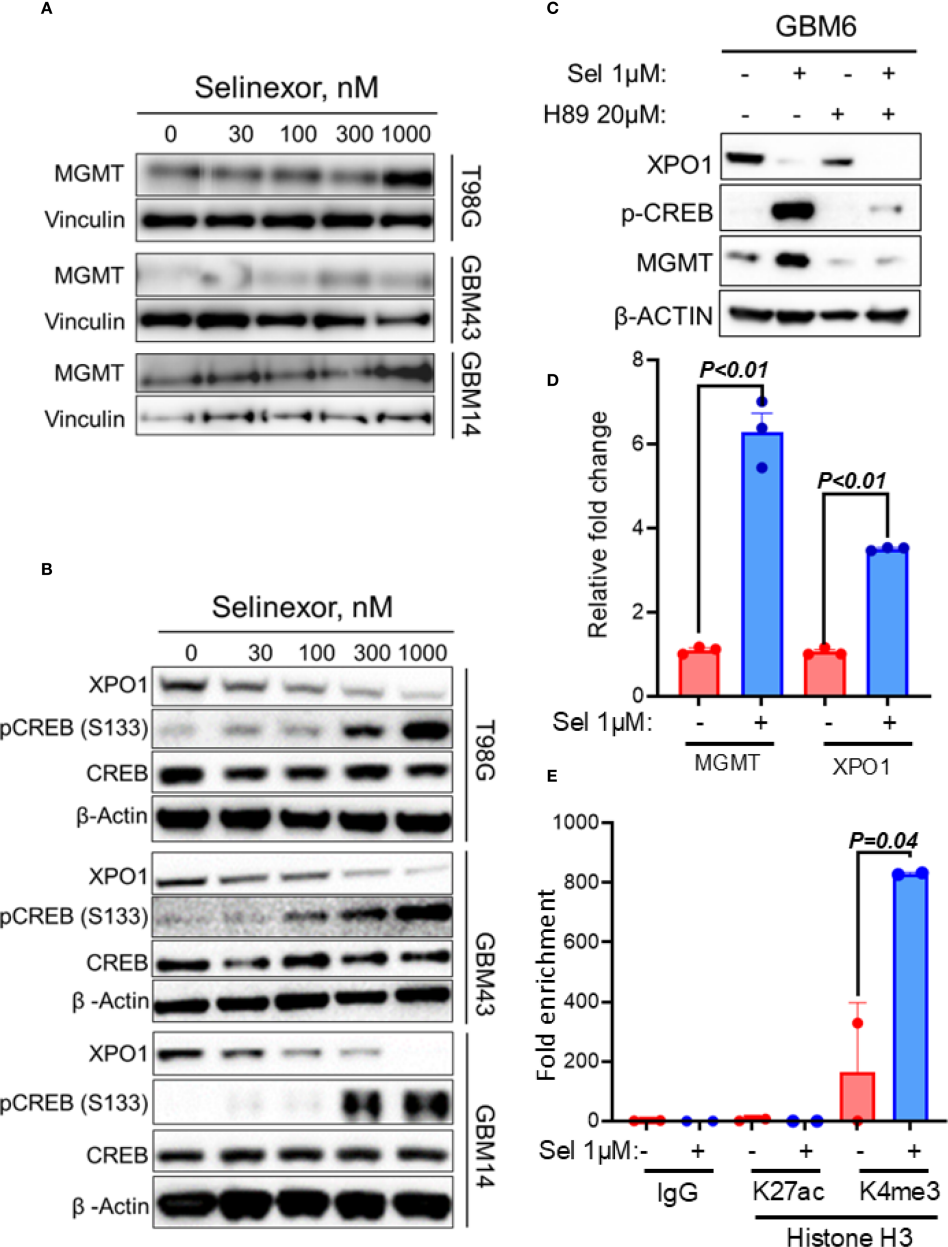
Effect of Selinexor on MGMT expression and phosphorylation of serine 133 of CREBB protein (p-CREBB^S133^). T98G, GBM43, GBM14 and GBM6, cells were treated with the indicated concentrations of Selinexor, and protein lysates were used for western blotting evaluation of **(A)** MGMT expression and **(B)** phosphorylation of serine 133 of CREBB protein (p-CREBB^S133^). **(C)** Western blotting showing the effect of PKA inhibitor H89 on Selinexor-induced MGMT and p-CREBB^S133^. **(D)** qRT-PCR displaying MGMT and XPO1 mRNA in GBM6 cells treated with the indicated concentration of selinexor. **(E)** The ChIP assay showed the increased trimethylation of lysine 4 of histone H3 (H3K4me3) within the MGMT promoter region of GBM6 when treated with Selinexor. Data in **(D, E)** are presented as mean ± SEM from n = 3 independent biological replicates, each performed in technical triplicates. Statistical significance was determined using a one-way ANOVA with Tukey’s multiple comparison test. Error bars represent SEM. Representative western blots in **(A–C)** are shown from n = 3 independent experiments.

As an initial step toward understanding the mechanism through which MGMT may control sensitivity to TMZ/Selinexor in GBM cells, we evaluated the effect of Selinexor on MGMT expression in unmethylated GBM cells. Interestingly, Selinexor strongly induced MGMT expression in unmethylated T98G and GBM14 cells. At the same time, less induction was observed in unmethylated low-MGMT GBM43 cells (**Figure 4A**). A qRT-PCR analysis showed increased MGMT transcripts and a rebound XPO1 mRNA in Selinexor-treated GBM6 (**Figure 4D**) and GBM14 cells (**Supplementary Figure S5**). Since the PKA-CREB pathway controls MGMT expression (28, 29), we tested whether Selinexor induces MGMT expression by activating this pathway. Indeed, Selinexor treatment blocked XPO1 protein in a concentration-dependent manner and was accompanied by increased phospho-CREB^S133^ in unmethylated GBM cells (**Figure 4B**). To confirm the role of the PKA-CREB pathway in mediating Selinexor-induced MGMT expression, we treated the unmethylated GBM6 cells with Selinexor with and without a PKA inhibitor, H89. As expected, Selinexor but not H89 suppressed XPO1 protein in GBM6 cells and was accompanied by increased MGMT and phospho-CREB^S133^ (**Figure 4C**). The PKA-CREB inhibitor H89 suppressed the basal and Selinexor-induced MGMT expression. Moreover, H89 significantly blocked the Selinexor-induced phospho-CREB^S133^ (**Figure 4C**). Next, we performed a qPCR to demonstrate whether Selinexor controls MGMT at the transcription level. As shown in **Figure 4D**, Selinexor treatment led to a 6-fold increase in MGMT mRNA in GBM6 cells. Since XPO1 mRNA was previously shown to be increased in Selinexor-treated tumors (30), XPO1 mRNA level was used as a positive control. Finally, we performed ChIP to establish the impact of Selinexor on the MGMT promoter chromatin accessible-histone status. As shown in **Figure 4E**, Selinexor increased the promoter decoration with trimethylated lysine 4 of histone H3 (H3K4me3) compared with the control DMSO-treated cells. Since the MGMT promoter is not decorated with H3K27Ac (https://genome.ucsc.edu/), we used this mark as a negative control. These findings suggest that selinexor induces MGMT transcription through PKA-CREB signaling-mediated promoter activation.

## Discussion

In this study, we investigated the role of XPO1 in TMZ resistance and delineated the potential of XPO1 inhibitor Selinexor in overcoming TMZ resistance in GBM cells. For the first time, we report that Selinexor induces MGMT expression by activating the PKA-pCREB^S133^ pathway. Moreover, we demonstrated that MGMT expression may determine sensitivity to a combined TMZ/Selinexor in GBM cells.

The current findings demonstrate that the XPO1 protein, a member of the nucleocytoplasmic (NC) pathway, confers TMZ resistance in GBM cells. The NC pathway is a critical cellular machinery that transports cargo from and into the nucleus and is largely controlled by a family of proteins known as karyopherins, including XPO1 and KPNA1 (importin-α1) (reviewed in (31)). XPO1 protein is an attractive target for cancer therapy because it is a nuclear exporter of oncogenes (e.g., CDKNA1, CDKNA2, and c-MYC), tumor suppressors (e.g., p53, p27, and Rb), and other growth-regulating proteins (e.g., FOXO3a, APC, NF-kB, and SURVIVIN) (14–16, 32). Accordingly, the XPO1 inhibitor Selinexor is an FDA-approved agent for treating refractory multiple myeloma (17, 18). A recent phase 2 recurrent GBM study demonstrated that Selinexor single-agent treatment could improve 6-month progression-free survival (22). However, because this study did not evaluate the sensitivity to combined Selinexor/TMZ treatment, the potential role of Selinexor in overcoming resistance in GBM patients after completing TMZ remains unelucidated. The present findings demonstrate that Selinexor can significantly resensitize resistant GBM cells to TMZ. Nonetheless, Selinexor treatment did not fully overcome TMZ resistance in GBM cells, which was not completely unexpected, partly because multiple mechanisms may be driving TMZ resistance in GBM cells. For example, a recent study suggests that the evolution of TMZ resistance is modulated by MGMT-dependent and -independent mechanisms in a cohort of promoter hypermethylated GBM PDX models (7, 23). In line with this view, besides the XPO1, this study identified an additional 141 candidate TMZ resistance genes, members of other pathways that require further elucidation by investigations beyond the scope of the current study. Interestingly, while this manuscript was under review another top candidate TLK1 (see **Figure 1C**) was reported to control TMZ resistance in GBM cells (33).

Selinexor was recently shown to enhance radiation in a preclinical model of orthotopic GBM tumors without a single-agent effect (34). However, knowledge about the sensitivity of GBM cells to a combined Selinexor/TMZ treatment, especially in the context of MGMT expression status, is lacking. The current *in vitro* findings demonstrated that MGMT-expressing GBM cells were sensitive to Selinexor single-agent treatment without the additional benefit when combined with TMZ. In contrast, MGMT-negative GBM cells were sensitive to the Selinexor single agent, and a significant additional growth suppression was observed in the Selinexor/TMZ combination treatment. These findings suggested that MGMT expression status may be a determinant of sensitivity to a combined TMZ/Selinexor treatment in GBM. These results closely resemble previous data demonstrating TMZ sensitization by PARP inhibition exclusively in MGMT promoter methylated GBM (35). Intriguingly, silencing MGMT enhanced, while forced MGMT expression blocked the sensitivity to combined Selinexor/TMZ treatment. Even though these findings may implicate MGMT in modulating sensitivity to combined selinexor/TMZ treatment, the mechanisms involved are subject to future investigations.

MGMT expression in GBM cells is largely controlled by epigenetic silencing through promoter hypermethylation in approximately 50% of GBM patients and is associated with a favorable TMZ response (6–9). Several other mechanisms are known to control MGMT expression in GBM cells. For example, several microRNAs have been shown to control MGMT expression in GBM cells (36, 37). Moreover, MGMT expression is controlled downstream of signaling pathways operating in GBM cells (38–40), suggesting that MGMT expression may be activated by the external stimuli targeting these pathways. The current study shows that selinexor treatment induces MGMT expression in parallel with the phosphorylation of serine 133 of the CREB protein (p-CREB^S133^). Since the PKA-CREB pathway was previously shown to control MGMT expression (28, 29), it is reasonable to conclude that Selinexor may increase MGMT expression through PKA-CREB signaling. Nonetheless, these findings should be interpreted cautiously, partly because the concentration of Selinexor that induced CREB phosphorylation was lower than the concentration that induced MGMT expression. Even though this may suggest that Selinexor may use different mechanisms to induce MGMT and p-CREB^S133^, this is unlikely because a PKA inhibitor abrogated both Selinexor-induced p-CREB^S133^ and MGMT expression. The present PKA-CREB findings, together with the previous studies reporting the activation of AKT (41), suggest that Selinexor may control the transcription of MGMT and other genes downstream to PKA-CREB and other pathways. Nonetheless, the mechanisms by which Selinexor activates the PKA-CREB signaling remain an interesting subject for future investigations.

In conclusion, these findings demonstrate that XPO1 plays a critical role in TMZ resistance and that Selinexor overcomes XPO1-mediated resistance in GBM cells. Moreover, MGMT promoter methylation status may be a useful determinant of sensitivity to combined selinexor/TMZ treatment, findings that await validation in patient samples from ongoing TMZ/Selinexor clinical trials. Since MGMT also plays a role in the development of acquired TMZ resistance in methylated GBM cells (23), the Selinexor/TMZ combination may enhance the evolution of MGMT-driven TMZ-resistance mechanism in GBM patients with methylated and unmethylated tumors. Therefore, combining TMZ with Selinexor may initially be beneficial only for newly diagnosed MGMT-promoter hypermethylated GBM patients.

## Data Availability

The original contributions presented in the study are included in the article/**Supplementary Material**. Further inquiries can be directed to the corresponding author.
