# Supplementary material for: XPO1-inhibitor Selinexor induces MGMT expression by activating PKA-CREB signaling in IDH wildtype glioblastoma
Source: Front Oncol. 2025 Sep 23;15:1633580. doi: 10.3389/fonc.2025.1633580 (PMC12500652; doi:10.3389/fonc.2025.1633580)
Supplement: Supplementary file 1 [file DataSheet1.pdf]

Supplementary Figures

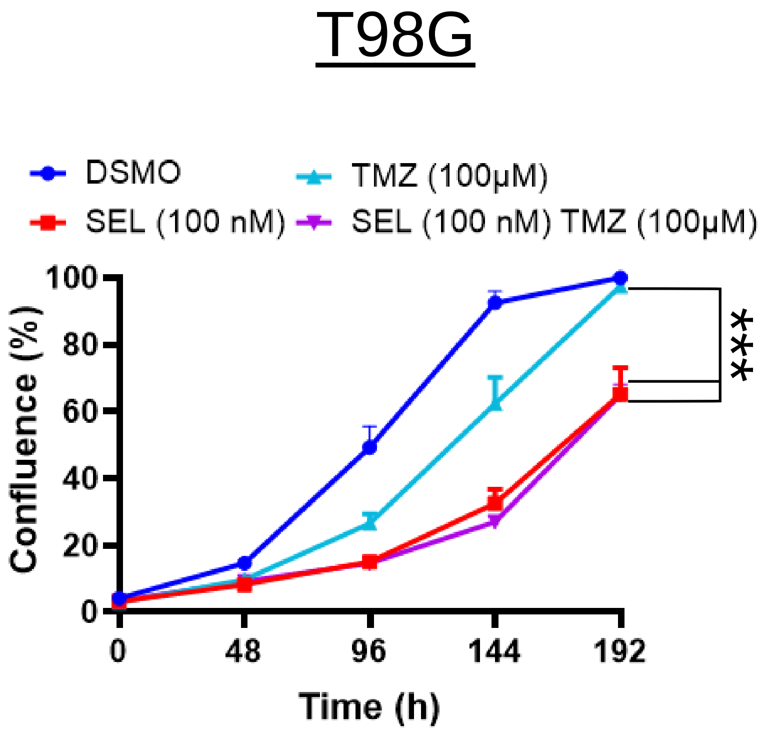

Figure S1: The sensitivity of MGMT-unmethylated T98G cells to Selinexor with and without TMZ. T98G GBM cells were treated with the indicated concentrations of Selinexor, TMZ, or Selinexor/TMZ. The control cells received the vehicle DMSO. *** $p<0.001$

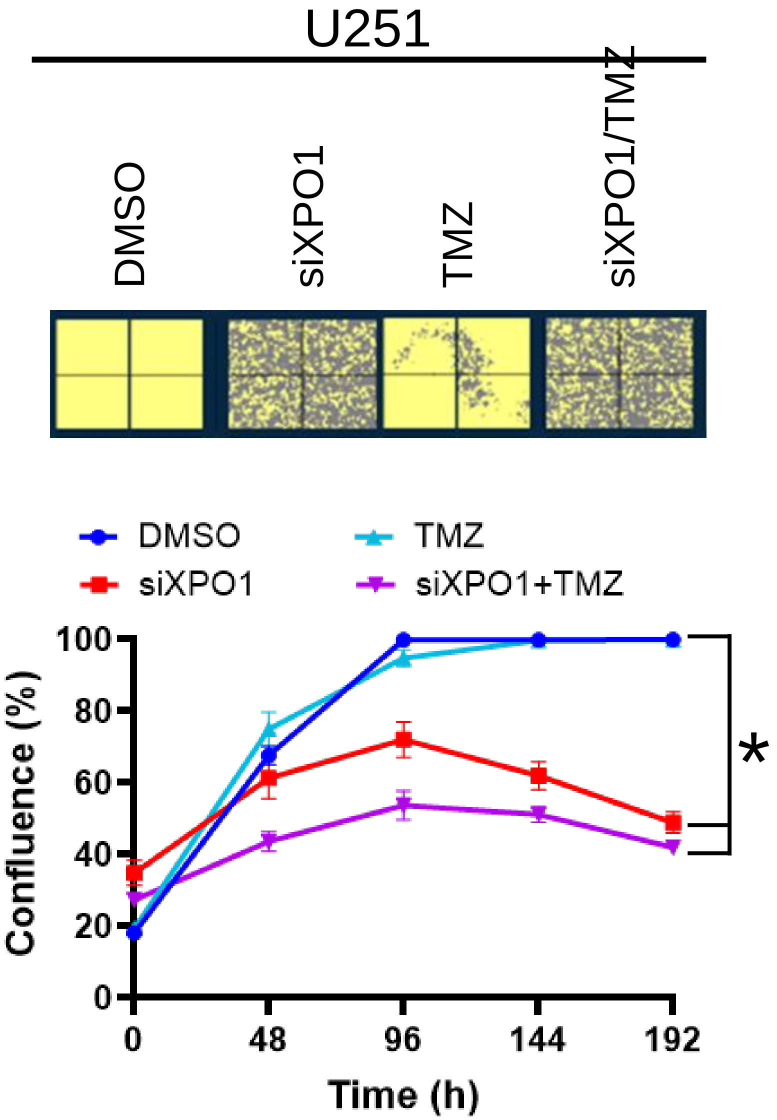

Figure S2: The effect of siRNA silencing on Selinexor with and without TMZ sensitivity in MGMT-methylated U251 cells. U251 cells were transfected with siRNA and treated with Selinexor, TMZ or Selinexor/TMZ. The growth of treated cells was live monitored in an Incucyte SX5 machine and reported as a percentage confluence. The confluence of cells masked in yellow is shown in the top panel. The lower panel displays the plots for each treatment group. * $p<0.001$ is shown in the top panel.

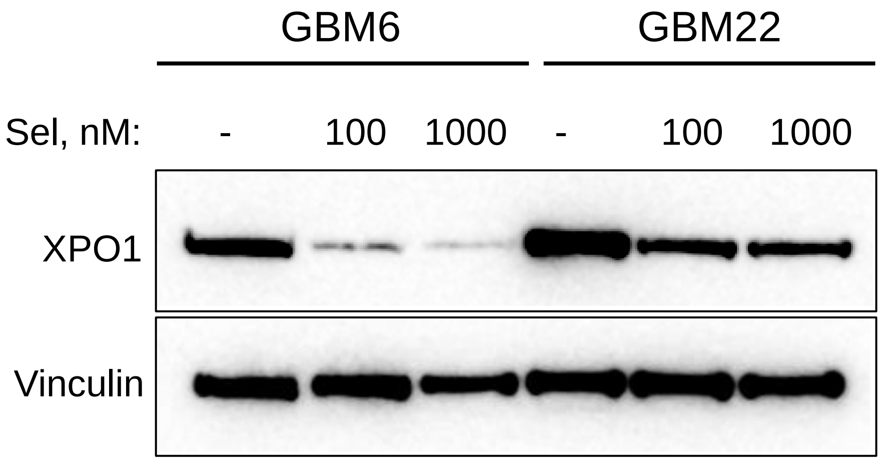

Figure S3: Effect of Selinexor on the expression of XPO1 protein. Cells primarily cultured from GBM6 and GBM22 PDXs were treated with and without Selinexor using the indicated concentrations and harvested 72 hours later. Vinculin was used for loading control.

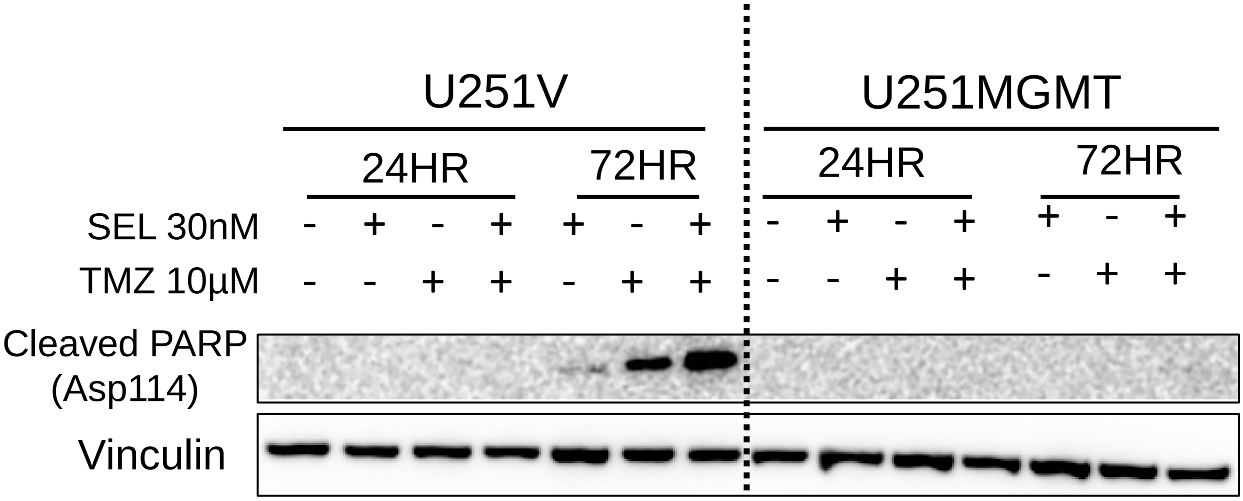

Figure S4: Effect of MGMT on apoptosis induced by Selinexor with and without TMZ. The MGMT-methylated U251 cells expressing empty vector (U251V) or MGMT (U251MGMT) were treated with the indicated Selinexor, TMZ, and Selinexor/TMZ and protein lysates were processed for western blotting 24 and 72 hours after treatment.

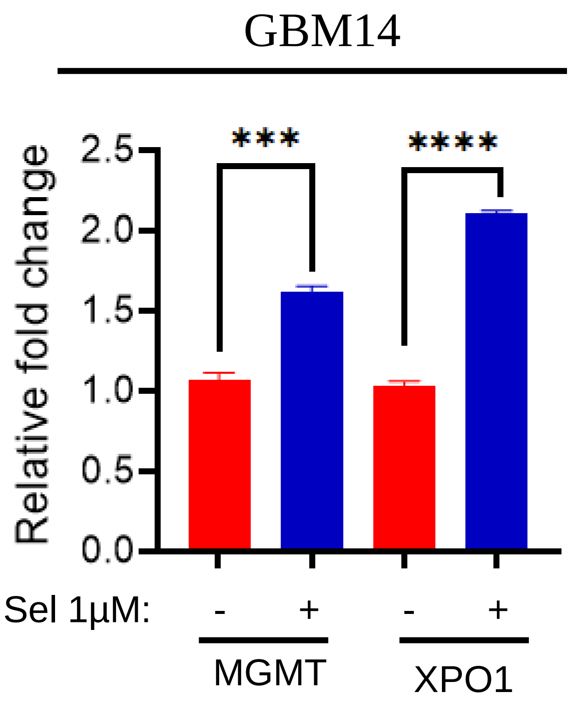

Figure S5: Effect of Selinexor on MGMT and XPO1 mRNA levels in primary cells cultured from GBM14. Cells were treated with the indicated Selinexor concentration, and total RNA extraction was performed 3 days later. The transcript levels were evaluated by quantitative real-time PCR in triplicate.
